# Progress and Prospects of Biomolecular Materials in Solar Photovoltaic Applications

**DOI:** 10.3390/molecules30153236

**Published:** 2025-08-01

**Authors:** Anna Fricano, Filippo Tavormina, Bruno Pignataro, Valeria Vetri, Vittorio Ferrara

**Affiliations:** 1 Dipartimento di Fisica e Chimica- Emilio Segrè, Università degli Studi di Palermo, 90128 Palermo, Italy; anna.fricano@unipa.it (A.F.); bruno.pignataro@unipa.it (B.P.); vittorio.ferrara@unipa.it (V.F.); 2 Dipartimento di Scienza e Alta Tecnologia and To.Sca.Lab, Università dell’Insubria, 22100 Como, Italy; filippo.tavormina@uninsubria.it

**Keywords:** photovoltaics, solar energy technologies, biomolecules, hybrid photovoltaic systems

## Abstract

This Review examines up-to-date advancements in the integration of biomolecules and solar energy technologies, with a particular focus on biohybrid photovoltaic systems. Biomolecules have recently garnered increasing interest as functional components in a wide range of solar cell architectures, since they offer a huge variety of structural, optical, and electronic properties, useful to fulfill multiple roles within photovoltaic devices. These roles span from acting as light-harvesting sensitizers and charge transport mediators to serving as micro- and nanoscale structural scaffolds, rheological modifiers, and interfacial stabilizers. In this Review, a comprehensive overview of the state of the art about the integration of biomolecules across the various generations of photovoltaics is provided. The functional roles of pigments, DNA, proteins, and polysaccharides are critically reported improvements and limits associated with the use of biological molecules in optoelectronics. The molecular mechanisms underlying the interaction between biomolecules and semiconductors are also discussed as essential for a functional integration of biomolecules in solar cells. Finally, this Review shows the current state of the art, and the most significant results achieved in the use of biomolecules in solar cells, with the main scope of outlining some guidelines for future further developments in the field of biohybrid photovoltaics.

## 1. Introduction

The growing need for sustainable energy solutions and the urgent environmental challenges related to extensive fossil fuel use are among the most pressing issues facing modern society. The widespread use of fossil fuels, such as coal, oil, and natural gas, has led to significant pollution and the release of greenhouse gases, which contribute to global warming and climate change. The detrimental effects on the environment have driven the research towards the development of new technologies to exploit cleaner and more sustainable energy sources [1]. Renewable sources of energy, such as solar, wind, and hydropower, have gained increasing attention thanks to their potential in solving many cons associated with the extensive use of fossil fuels [2]. Among renewable sources, solar energy is likely the most promising. Indeed, the Earth receives in one hour approximately 173,000 TWh of energy from the Sun as solar radiation, an amount slightly exceeding the total annual human energy consumption [3]. Also, solar energy can be harnessed in various ways, such as converting sunlight into electricity using solar panels, heating water, or powering large-scale solar power plants. Therefore, solar energy technologies have advanced rapidly in the last decades and today represent the main global shift toward renewable energy. The most studied and applied methods for capturing solar energy are based on the photovoltaic effect and light harvesting process. The photovoltaic effect consists of the polarization of a photoactive system, typically a semiconductor p-n junction, upon exposure to radiation of a proper range of wavelengths. The fundamental principle behind the photovoltaic effect consists in the absorption of photons exciting electrons from the valence band to the conduction band in a semiconductor. Excited electrons and the corresponding holes are then separated by the built-in electric field proper of the p-n junction, resulting in a polarization of the system. The polarized system can be connected to an external circuit to get a photogenerated current. Such an effect is fundamental for solar cells to directly generate electricity as exposed to sunlight or artificial light sources in indoor applications. On the other side, light harvesting focuses on using sunlight to drive photochemical reactions, typically through collection and transfer of solar energy by photoactive chromophores or their macromolecular complexes, such as in biological photosynthetic systems [4]. Upon photon absorption, energy transfer can occur among electronic excited states of chromophores towards reaction centers, where the energy is exploited to drive chemical reactions. Therefore, the photovoltaic effect directly converts light into electricity through an optoelectronic transduction system; meanwhile, light harvesting focuses on light absorption and energy transfer, often as part of a more complex physicochemical process involving photochemical reactions. In both cases, great care must be taken in selecting materials. To exploit the potentiality of sun energy and fabricate durable devices. For instance, silicon-based solar panels have reached mirable records of about 27% in efficiency, approaching the theoretical limit of ~30%, and more than 20 years of certified durability.

To provide context for the integration of biomolecules into solar cells, this Review begins with a concise overview of the evolution of photovoltaic (PV) technology, tracing its historical development both in terms of materials and architectures. A comparison of the various solar cell generations is outlined, based on the materials used, and the most significant improvements obtained are described, as well.

By analyzing recent developments reported in the literature, particularly in the field of organic photovoltaics, and the incorporation of biomolecules into solar energy conversion systems, this Review aims to go beyond a simple overview of the emerging technologies. It would offer a critical assessment of the current state of the art in bio-hybrid solar cells, drawing attention to both the most significant breakthroughs and the persistent challenges that still limit their widespread application. Special focus is placed on how biomolecules, such as proteins, peptides, DNA, and other biological components, have been explored as active or supporting elements in light-harvesting and charge transport systems. This Review is structured to highlight the added value that biomolecules can bring to photovoltaic devices, including their potential for enhancing light absorption, improving interface properties, or enabling self-assembly and structural control at the micro- and nanoscale. At the same time, it critically addresses the limitations and bottlenecks associated with their use, such as stability under operational conditions, integration with synthetic materials, and scalability. Therefore, the Review intends to offer a comprehensive overview of the current state of the art, but also a set of conceptual and practical guidelines to help researchers navigate this complex and rapidly evolving area. This includes outlining promising directions for future investigations, such as the design of hybrid materials with improved long-term stability, or the development of new architectures that exploit the functional versatility of biomolecules. Ultimately, this Review is conceived as both a reference and a strategic guide for scientists and engineers working at the intersection of biology and solar energy technologies. It seeks to support informed decision-making in experimental design and material selection, while also fostering a deeper understanding of the opportunities and limitations that come with incorporating biomolecular systems into next-generation photovoltaic devices.

## 2. Evolution of PV Technology

### 2.1. Solar Cells Generations and Advancements

Photovoltaics generate electricity directly from sunlight, and PV technology has advanced rapidly in recent years, reaching a crucial role for the development of future energy production [5,6]. The photovoltaic effect was first discovered by Alexandre-Edmond Becquerel in 1839 [7], later applied by the American inventor Charles Fritts to develop the first solid-state solar cell prototype, by using selenium as photoactive phase. Then, the field was explored deeply, reaching successful goals which resulted in solar panels based on silicon widely installed in many natural and urban areas, such as PV fields and on building roofs.

The development of modern PV technology began in 1954 with the fabrication of first-generation solar cells. The first-generation cells mainly consist of solar cells with silicon-based p-n junction as photoactive phase. Silicon solar cells showed an initial power conversion efficiency (PCE) of 6%, later improved to 14% under natural sunlight. In the 1980s and 1990s, silicon-based solar PV has become the dominant PV technology on the market, thanks to the high PCE with a current record of 27%, reliability, and long lifespan, guaranteed for 40 years. Silicon solar cells on the market can be distinguished in monocrystalline, offering the best performance in terms of photoconversion efficiency, and polycrystalline, which represents a good compromise between efficiency and cost [8,9].

Indeed, the main limitation to the widespread use of silicon PV is the cost. Anyway, recent market trends show a remarkable decrease in the cost of solar panels, with high-efficiency modules in Europe reaching as low as €0.13/Wp by late 2024, that is a 43.5% drop compared to the beginning of the year [10]. This sharp decline, largely driven by global oversupply and reduced production costs, has significantly enhanced the economic feasibility of photovoltaic systems, particularly for residential installations. Also, costs are still decreasing in recent years, making solar energy always more accessible for renewable energy production.

Second-generation cells introduce the use of thin-film semiconductors to reduce the amount of materials and costs. While monocrystalline and polycrystalline silicon cells remain dominant, research on thin-film solar cells has grown to offer a more cost-effective alternative with high efficiency. Examples include amorphous silicon (a-Si), cadmium telluride (CdTe), and Copper Indium Gallium Diselenide (CIGS) thin-film cells. Although less common, these are also available commercially and fall under the second generation of solar cells.

Third-generation solar cells aim to surpass the costs and some applicative limitations of previous technologies by using innovative materials and designs. Unlike first generation (silicon-based) and second-generation (thin-film) cells, third generation cells explore the use of several innovative kinds of materials and cell architectures such as perovskite solar cells in pin and nip configuration, quantum dots, dye-sensitized solar cells, semiconducting polymers, and the concept of planar and bulk multi-junction solar cells. These technologies offer valuable efficiency combined with the potential for lower manufacturing costs. In particular, perovskite solar cells have gained significant attention due to their rapid efficiency improvements reaching record PCE of 26% comparable with silicon solar cells. Quantum dot solar cells exploit nanoscale semiconductor properties to enhance and tune light absorption. Dye-sensitized solar cells (DSSCs) use photosensitive dyes to absorb sunlight and generate electricity. Organic PV is mainly based on the use of semiconducting polymers combined in planar and bulk heterojunctions towards the development of cheap and flexible classes of solar devices. The chemical precursors of all the mentioned materials used as absorbers in third generation solar cells are readily soluble in common organic solvents allowing high volume throughput manufacturing techniques as roll-to-roll (R2R), and inkjet printing, highly valued for the knowledge on the fluid dynamics principles controlled to tune the features of deposited materials, as well as for the scale up of the fabrication protocols at the industrial level [11,12]. Among the plethora of interesting peculiar features of each kind of third generation solar cells aforementioned, these innovative PV systems have stood out not only for their performance in terms of photoconversion, but also because they introduce new fields of application for solar technology not accessible to silicon-based technologies. For instance, organic and perovskite solar cells can be fabricated to obtain semi-transparent devices that can be used as photovoltaic windows, devices’ screens that contribute to powering the device itself, or coupled with other devices, even with the silicon ones, in tandem configuration by properly tuning the band gaps and thus the light absorption range of each paired device. The tandem configuration is of great interest since it offers the possibility to overcome the intrinsic limit of photoconversion efficiency that characterizes every material. These aspects can open up totally new applications for PV technologies. Although the third generation solar cells still face challenges in stability, scalability, and commercialization, the research continues towards improving their performance, making them promising candidates for the future of renewable energy [13,14].

**Figure 1:**
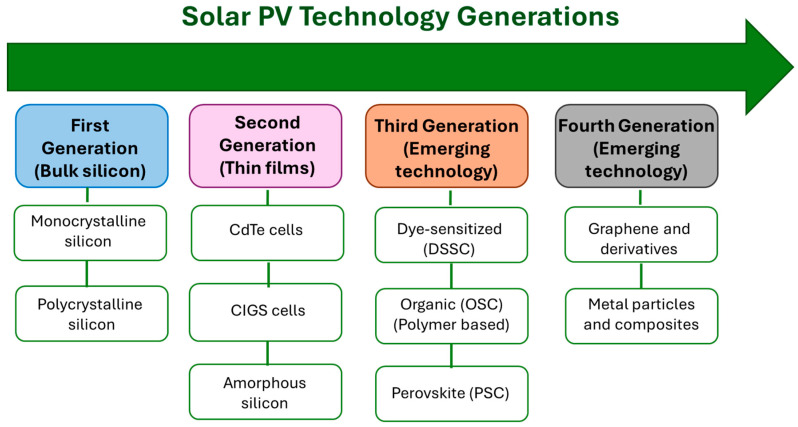
Schematic illustration of fourth generations of PV cells [23]. First-generation solar cells, commonly referred to as conventional or traditional solar cells, are primarily composed of silicon and their efficiency has ranged from 6% to 15% has significantly improved, reaching up to 25%. Second generation solar cells, also known as thin-film solar cells, are made from materials like copper indium gallium selenide (CIGS), cadmium telluride (CdTe), and amorphous silicon (a-Si). They are thinner than traditional solar cells and have an efficiency range of 10–15%. Third-generation solar cells utilize materials that are cheap and abundant, such as perovskites, dye sensitized solar cells, and organic solar cells, which can be processed using low-cost manufacturing techniques. Recent advancements have introduced the use of biomaterials, which enhance sustainability and potentially improve energy conversion efficiency. Fourth-generation solar cells incorporate innovative materials such as graphene and metal nanoparticles commonly in organic-inorganic composites.

Recently, a new class of solar cells has emerged from the effort to employ innovative materials combining the advantages of previous generations [15,16]. These new devices form fourth-generation solar cells. This generation is based on a careful combination of organic and inorganic materials, often in the form of composites [17]. Although still at an early stage of development in terms of integration of biological components within their architectures, fourth-generation solar cells can represent a further field to explore for future advancements in bio-hybrid solar technologies. As an example, it is possible to infer that loading photoactive components in noble metal nanoparticles composites can constitute an interesting strategy in order to exploit the combination of plasmonic properties with photovoltaic behaviour towards an improvement in terms of device efficiency. Typical materials of this generation include metal oxides and metallic nanoparticles, as well as organic nanomaterials such as graphene, carbon nanotubes, and their derivatives, and many others 2D materials such as molybdenum disulphide (MoS_2_), graphene, tungsten disulphide (WS_2_) and tungsten diselenide (WSe_2_), often combined into composites characterized by flexibility and lightweight. Due to the use of such materials often combined in solid state composites, this class of chemical systems employed in fourth-generation PV devices is commonly referred to as inorganics-in-organics. Some examples of this approach have been reported by Pignataro and coworkers, who developed physicochemical strategies for the fabrication of composites based on metal nanoparticles embedded in polymeric matrices [18,19]. A first example involves the incorporation of TiO_2_ nanoparticles into an in situ formed polysiloxane matrix, with a control on the dispersion and clustering of TiO_2_ nanoparticles by applying thermal treatment at the solvent’s boiling point [18]. A second example concerns the use of gold nanoparticles (AuNPs) functionalized with thiol groups and embedded at the planar heterojunction between poly(3-hexylthiophene-2,5-diyl) (P3HT) and [6]-phenyl-C_61_-butyric acid methyl ester (PCBM) in organic solar cells. This configuration led to a 14-fold increase in power conversion efficiency, attributed to localized light scattering in the critical working region of the device, which enhanced light absorption and charge collection [19]. The different types of 2D materials mentioned above have been explored in the development of solar cells due to their versatility with different roles such as transparent electrodes, electron or hole transport layers, active layers, buffer layers, or even as ultrathin, transparent diffusion barriers. Among the most interesting examples, the use of 2D transition metal dichalcogenides has demonstrated significantly enhanced light absorption, achieving absorption rates of 5–10% with material thicknesses under 1 nm [20] monolayer MoS_2_ can be integrated with p-type silicon to obtain heterojunction solar cells, as well [21]. Also, graphene is getting widely utilized in photovoltaic devices thanks to its tightly packed, 2D honeycomb lattice, which also offers protection against environmental degradation [22]. The different solar cell generations and their temporal evolution are schematically reported in Figure 1.

The rapid evolution of photovoltaics in terms of materials, device architecture, and fabrication techniques reflects the advancements observed from one generation to the next. The different generations are not intended to replace the previous ones; all of them are of interest for both research and industry because of their own distinctive features, making them suitable for specific applications in different contexts. For example, first-generation silicon-based cells, known for their high efficiency and stability, are commonly used in large-scale photovoltaic farms and rooftop installations. Second generation thin-film technologies, which are lighter and more flexible, are ideal for integration into building facades, portable devices, or curved surfaces. Third-generation cells, including perovskite and organic photovoltaics, offer advantages like semi-transparency and tunable color, making them suitable for use in smart windows, wearable electronics, and even solar-powered sensors embedded in everyday objects.

#### Solar Cells: General Aspects

Different types of solar cells have been outlined in the previous selection, highlighting the peculiar aspects of each generation. However, some general fundamental principles can be defined for typical solar devices in terms of structure and basic physics of charge carriers. In fact, a modern solar cell can be generally described as a system where a photoactive layer, responsible for light absorption and free charges generation, is sandwiched in between transport layers and electrical contacts. The transport layers, typically electron and hole transport layers (ETL and HTL, respectively), facilitate movement of charge carriers toward the electrodes, which collect and allow the charges to flow into an external circuit, generating electrical power. This electrically symmetric configuration enables effective charge separation by directing electrons toward the cathode and holes toward the anode. This efficient routing of charge carriers is essential for minimizing recombination losses and maximizing the overall performance of the solar cell.

**Figure 2:**
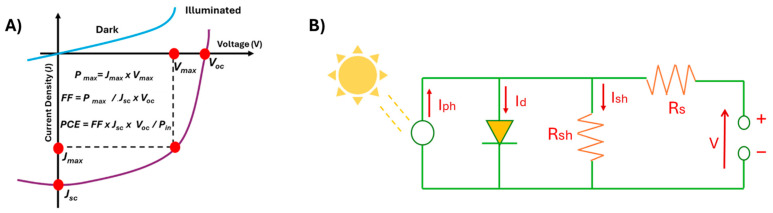
(**A**) A typical current density– voltage (J–V) curve of OSC devices and the meaning of J_SC_, V_OC_, FF, and PCE. The J–V curve represents the relationship between the current density (J) and the applied voltage (V) of a solar cell under illumination. The arrows indicate the current flow for each component in the equivalent circuit. Short-circuit current density (J_sc_) represents the maximum current the solar cell can deliver under standard illumination conditions. Open-circuit voltage (V_oc_) indicates the maximum voltage the device can provide, related to the energy difference between the conduction and valence bands. Fill factor (FF) indicates the “squareness” of the J–V curve. Higher FF values suggest lower internal resistive losses and better device quality. PCE measures how efficiently the solar cell converts sunlight into usable electrical energy [24,25]. (**B**) Equivalent circuit of a solar cell under illumination. The incident sunlight generates a photocurrent (I_ph_) modeled by a current source. The diode represents the junction behavior, while the series resistance (R_s_) and shunt resistance (R_sh_) model resistive losses and leakage pathways, respectively.

Some key parameters provide a comprehensive evaluation of solar cell performance. They offer a clear and systematic understanding of how a device operates and are essential for comparing different technologies and materials. All the parameters that describe the fundamentals of a solar device can be either graphically derived or calculated from the measurement of the current at different potential values, namely I-V characteristic. A schematic of an I-V characteristic for a solar cell is shown in Figure 2, where the most important parameters are highlighted. First of all, this graph immediately allows us to define the behavior of the system as typical for a solar cell, as the I-V characteristic of a solar cell exhibits the typical shape of the curve reported. From a mathematical perspective, the IV characteristic of a solar cell is modeled as an exponential, which relates the relationship between current and voltage, taking into account factors like saturation current, ideality factor, and series and shunt resistances. The parameters describing the solar cell features are mainly the following. Short-circuit current density (J_sc_) indicates the current density at 0 V, and represents the maximum current the solar cell can generate under standard illumination conditions. The open-circuit voltage (V_oc_) of a solar cell is the voltage measured as no current is flowing through the external circuit. It is related to the value of the band gap and is affected by factors such as the quality of the junctions and recombination processes within the cell. The fill factor (FF) is a parameter that characterizes the efficiency of a solar cell. It is defined as the ratio of the area calculated as the product of the J_max_ and V_max_ to the product of the V_oc_ and the short-circuit current (I_sc_), which represent the theoretical and experimental maximum power a solar cell can produce. The FF provides insight about the internal resistances in the device. A high FF indicates that the solar cell operates closer to its theoretical maximum power, with minimal losses due to resistance or recombination. PCE is the ratio of the electrical power output and measures how efficiently the device converts sunlight into electrical energy, expressed as a percentage. The formula for PCE is:

$$PCE=(FF\times V_{oc}\times J_{sc})/P_{in}$$

This equation expresses the efficiency of a solar cell by considering the open-circuit voltage (V_oc_), short-circuit current density (J_sc_), fill factor (FF), and the incident power (P_in_), this is the power density of the light hitting the solar cell, typically standardized at 100 mW/cm^2^ (1 sun), all of which determine how effectively the cell converts sunlight into electrical power relative to the incident solar energy. With all these parameters, obtained from a simple characterization, it is possible to compare different solar cells by using a unified approach, allowing for the evaluation of their electrical performance. This comparison provides insights into the dynamics of charge carriers and the energy bands involved, offering valuable information for further optimization of materials and junction interfaces.

The cell parameters can be related to the equivalent circuit of a solar cell, which helps to better figure out the properties and behavior of a solar device from an electrical point of view. As shown in Figure 2B, a typical equivalent circuit of a solar cell includes an ideal current source, which corresponds to the solar device, in parallel with a reverse-bias diode, connected to a load. The ideal current source (I_ph_) in the equivalent circuit corresponds to the photogenerated current, which is directly proportional to the incident light intensity. This component is associated with the I_sc_, representing the maximum current output when no voltage is applied. The diode in parallel accounts for the internal recombination behavior within the cell and is linked to the V_oc_, which depends on the balance between photogenerated and recombination currents. The output power, product of current and voltage, depends also on the relative resistance of the load, modeled by using series and shunt resistances. Shunt resistance (R_sh_) is parallel to the current source and represents all losses that result in the charge carriers’ path between the electrodes. The R_sh_ represents leakage paths due to defects or internal short circuits; then, low R_sh_ values decrease I_sc_ and maximum power output (P_max_), and influence the I-V curve slope at low voltages. Series resistance (R_s_), in line with the current path, accounts for poor charge transport and must be minimized to maintain efficient current flow. The R_s_ models ohmic losses during current transport through the device, such as those caused by contacts or active layers. High R_s_ values reduce both current and P_max_ and affect the slope of the I-V curve at high voltages.

### 2.2. Third Generation Solar Cells: Design Principles, Material Innovations

**Figure 3:**
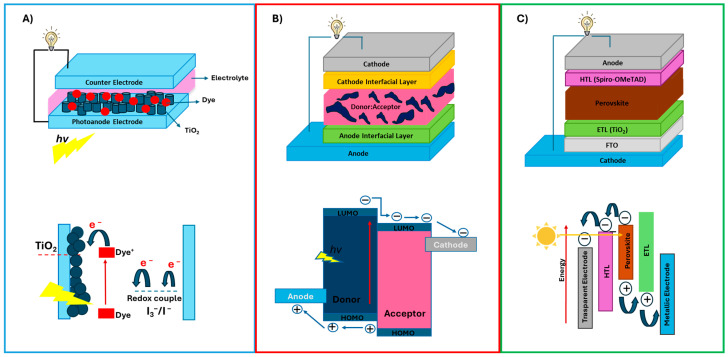
Schematic views of common architectures with the corresponding working mechanisms: in (**A**) DSSC consists of a dye-sensitized photoanode, a redox electrolyte, and a counter electrode in which incident light excites dye molecules, which inject electrons into the TiO_2_ conduction band; the oxidized dye is subsequently regenerated by the electrolyte; (**B**) bulk heterojunction OSC features a donor and acceptor materials, positioned between interfacial layers and electrodes, photoexcitation generates excitons in the donor material, which then dissociate at the donor–acceptor interface, allowing charge carriers to reach the electrodes; (**C**) Perovskite solar cells (PSCs) are composed of a multilayer structure in which the perovskite layer absorbs light and generates charge carriers that are efficiently separated and transported to the electrodes through dedicated electron and hole transport layers (ETL and HTL). The arrows in the diagram represent the direction of charge generation, transport, and collection in three types of photovoltaic devices.

Third-generation solar cells appear to be the most promising in terms of biomolecule integration. For this reason, their characteristics will be further explored in terms of materials and architectures. The most common architecture and the corresponding working mechanisms are schematically represented in Figure 3. Firstly, organic solar cells (OSCs) attract attention for biomolecule integration, due to their chemical affinity with the biomolecules characterized by organic nature themselves. OSCs generate electric current from sunlight through the excitation of electrons in a polymeric semiconductor. This occurs in OSCs when electron excitation occurs in the photo-active layer, typically composed of a conjugated polymer and a fullerene derivative, or other acceptor material. Photons with sufficient energy excite electrons from nearly full energy levels to mostly empty ones, resulting in an electron-hole pair, namely, an exciton. Due to strong Coulombic attraction in organic semiconductors, these excitons have to reach the donor-acceptor interface for the charge separation to occur. The electron transfers to the acceptor, while the hole moves into the donor material, both generating free charge carriers that contribute to the photocurrent. Both electrons and holes are free to move within the solar cell, but they have a limited lifetime as they can lose energy through many different physical and chemical mechanisms, leading to less efficient photo-conversion in solar devices. The key goal is to identify organic materials which can better perform in terms of light absorption and charge to exploit the advantages of organic devices in real applications. Indeed, the use of molecular semiconductors, such as conjugated polymers or small organic molecules, rather than traditional inorganic materials like Silicon or Gallium Arsenide, is the most peculiar and interesting aspect for organic solar cells. This distinction offers unique advantages in flexibility, cost, and potential for large-scale production [23].

An OSC is composed of several functional layers. First, OSCs are typically fabricated either on glass or plastic substrates since the fabrication protocols for these kinds of devices are compatible with organic flexible materials. Such substrates are coated with a bottom electrode, for example, the indium tin oxide (ITO), which represents a common highly transparent conductive layer, a useful aspect for applications of OSCs in building integrated PV and tandem configuration. An anode interfacial layer is then coated on the bottom electrode, such as PEDOT:PSS, as a common choice since this material enhances hole injection and extraction, and can be processed using liquid deposition techniques, including as an aqueous solution. Then, the active phase is deposited, which represents the fundamental working component of the cell. A commonly used photo-active material is P3HT, a widely studied p-type semiconducting polymer in organic solar devices due to its ease of processing, low cost, and compatibility. In particular, P3HT exhibits notable hole mobility, but limited stability under ambient conditions, primarily due to the low ionization potential of the thiophene units in its backbone, which makes it susceptible to degradation [26].

The light-absorbing active layer can be deposited in a planar heterojunction configuration in between the anode and cathode interfacial layers, or as a mix of the photo-active material and an acceptor compound, to form a single-layer bulk heterojunction (BHJ) (See Figure 3). BHJ in polymer solar cells is increasingly recognized as a promising renewable energy technology due to its simple fabrication, low production cost, lightweight design, and potential for flexible device applications. In these cells, the active layer consists of a blend of electron-donating materials (p-type conjugated polymers) and electron-accepting materials (n-type fullerene derivatives). A well-studied example of BHJ PSCs is the combination of P3HT as the electron donor and PCBM as the electron acceptor [27]. To complete the structure, high-work-function metal top electrodes, commonly made from aluminum, silver, or gold, are added, along with encapsulation layers that protect the cell from environmental degradation [28].

Despite reaching efficiencies as high as 19.2%, OSC technology still faces several limitations. One major challenge is the relatively short lifespan of organic materials, which can degrade over time when exposed to light, heat, or oxygen. Additionally, OSCs often have lower stability and performance compared to other solar technologies, and improving the charge mobility and reducing energy losses remain ongoing challenges. Lastly, large-scale manufacturing and cost-effectiveness continue to be areas requiring further development to make OSCs more commercially viable. To improve stability, researchers have investigated modifications such as adjusting the polymer’s geometry, altering side chain regiochemistry, and developing copolymers with thiophene units. These changes aim to increase the material’s ionization potential, thereby enhancing air stability. Advances in this field have led to the development of more stable p-type polymers that maintain performance under ambient and even harsher conditions. Another promising approach involves the use of all-polymer solar cells, specifically blending the polymer P(NDI2OD-T2) with P3HT. Notably, this blend has achieved a high FF of nearly 70% for the first time, indicating well-balanced charge mobility within the polymer-blend thin films. The high electron mobility of P(NDI2OD-T2) enables FF values comparable to those reported for fullerene-based devices [29]. Through various optimization techniques, including thermal treatment, solvent, and vapor annealing, as well as mixed-solvent approaches, the PCE of P3HT PSCs has surpassed 4%. These treatments enhance the morphology and phase separation within the active layer, which is critical for improving charge separation and transport, ultimately boosting the efficiency and stability of the devices.

Inspired by natural photosynthesis, O’Regan and Grätzel made significant strides in DSSCs in 1991, achieving efficiencies of nearly 8%. DSSC consist of four main components: a photoanode, which is a porous layer of TiO_2_ (or ZnO) nanoparticles on a glass substrate with a transparent conducting film, a monolayer of dye molecules adsorbed on the TiO_2_, a back electrode on another glass substrate with a transparent conducting film, and an electrolyte layer, typically an iodine-based redox couple, sandwiched between the two substrates [30].

Unlike traditional solar cells, which both absorb light and transport carriers in the same semiconductor material, DSSCs separate these functions. The dye molecules absorb photons, and the TiO_2_ transports the excited electrons. The electrons flow through the TiO_2_ to the back contact, while the electrolyte restores the oxidized dye molecules to their original state. These cells can be partially transparent, flexible, and are cheaper to produce than conventional solar cells due to their simple materials and fabrication. Natural dyes offer additional cost advantages, as they are abundant, eco-friendly, and require less complex extraction methods compared to synthetic dyes. DSSCs are less affected by material impurities, making them cheaper to produce than monocrystalline silicon solar cells [31]. A few years later, OSCs emerged with an initial efficiency of around 1%, but by the early 2010s, they surpassed DSSCs, now reaching efficiencies of up to 18% [32].

Perovskites, first discovered in 1978 by Weber and later explored for light-emitting applications, evolved from DSSCs [32,33]. A typical PSC consists of a multilayer constituted by the perovskite deposited between the ETL and HTL. A bottom and a top electrode allow for electrical connection to the external circuit. In perovskite solar cells, the working mechanism begins with photoexcitation, where incident sunlight is absorbed by the perovskite layer. Photons with energies equal to or greater than the bandgap excite electrons from the valence band to the conduction band, creating electron-hole pairs. Due to the low exciton binding energy in perovskites, these pairs rapidly dissociate into free charge carriers even at room temperature. Electrons and holes are separated and transported to their respective selective contacts, ETL often made of materials like TiO_2_ or SnO, and HTL such as spiro-OMeTAD or PTAA, respectively. Efficient transport is facilitated by the favorable energy level alignment and high carrier mobility of the perovskite, leading to charge extraction at the electrodes and the generation of photocurrent [34].

Although third-generation solar cells offer several benefits, they still have limitations, in particular stability concerns mainly associated with their sensitivity to water and oxygen from air, as well as to physical stimuli such as light and heating. However, these challenges represent opportunities for advancement, especially through the design of new materials and fabrication strategies. To this aim, recently biomolecules have emerged for photovoltaic applications as candidates to screen for powering solar cells’ performances and durability. Biomolecules, such as nucleic acids, proteins, pigments, and enzymes, are being integrated into solar cells to enhance their efficiency and sustainability. For instance, researchers are exploring photosynthetic proteins and light-harvesting complexes from plants and algae to create biohybrid solar cells. These biomolecules can improve light absorption and energy conversion processes, potentially leading to more efficient solar panels. Recent studies have also focused on combining biomolecules with traditional photovoltaic materials, to create more stable and efficient solar cells. These hybrid systems take advantage of the unique properties of biomolecules while benefiting from the performance of synthetic materials widely used for modern solar cells [35].

## 3. Introduction to Biomolecular Materials in Solar Applications

Integrating biomolecules into the photovoltaic field offers a significant opportunity for innovation and aligns well with the principles of the circular economy, inspired by natural systems where resources are continuously reused, and waste is minimized [36]. Indeed, biomolecules can offer significant potentiality to enhance the energy efficiency, durability, and biodegradability of solar cells, contributing to the development of more sustainable and environmentally friendly solar panels [37]. However, the integration of biomolecules into PV devices is not straightforward at all. On the one hand, integrating biomolecules is advantageous since numerous categories and biomolecular structures are available for exploring their use in solar technology, offering a wide range of possibilities to improve the different functional layers in PV devices. On the other hand, biomolecules are often challenging to use in this context, especially because of the presence of adsorbed water molecules, which are difficult to fully eliminate and can compromise the performance and stability of most of the solar devices. Therefore, in order to fully exploit the advantages of using biomolecules in solar applications and to optimize their integration into devices, a systematic approach based on a rational strategy is mandatory to investigate the application of such a huge variety offered by biomolecules.

### 3.1. Roles of Biomolecules in PV

Biomolecules constitute intriguing systems which can be exploited to design materials and composites at the interface between biology and material science, composed of complex molecular structures that exhibit versatile electronic, optical, and self-assembling properties, making them promising candidates for enhancing solar energy conversion [38].

Biomolecular materials can serve multiple functions in solar cells, taking on both active and passive roles. Active roles mainly include functioning as photoactive phases, charge transport layers, while passive roles involve providing structural support, ecological substrates, and insulating encapsulation layers.

As active materials, some biomolecules have emerged for their remarkable ability to absorb light across a wide range of wavelengths, making them highly versatile for energy applications. One of their most intriguing features is their tunability, which stems from the capacity to alter their molecular structure. By modifying these structures, scientists can precisely control the materials’ optical and electronic properties, tailoring them for specific functions. A prime example of this is photosynthetic proteins found in nature. These proteins are highly efficient at capturing and transferring solar energy within plants and microorganisms, serving as a model for designing synthetic solar cells. By mimicking the mechanisms of these natural proteins, researchers have been able to develop solar cell technologies that achieve enhanced performance and efficiency.

In addition to their absorption and tunability, biomolecules exhibit an extraordinary ability to self-assemble into highly organized, hierarchical structures. This self-assembly mirrors the sophisticated arrangements found in natural light-harvesting systems, such as those in photosynthetic organisms. These well-ordered structures naturally facilitate the efficient movement of electrons, creating optimized pathways that are critical for effective energy conversion. By replicating these natural systems, biomolecular materials can significantly improve the performance of artificial light-harvesting devices [39]. To enable their optimal use in optoelectronic devices, the interactions between commonly used device materials and biomolecules must be thoroughly investigated at the molecular level. Processes of adsorption and changes in structure for the biomolecules upon interaction with synthetic materials are fundamental to be taken into account to exploit the proper function of biomolecules in optoelectronics [40]. Attention has to be paid to these aspects since photoactivated side processes can lead to damage and changes in biomolecules, particularly as they are deposited at the interface with photocatalytically active semiconductors such as TiO_2,_ commonly used as ETL in photovoltaics. It has been demonstrated that TiO_2_ can induce structural modifications in biomolecules, potentially compromising their function [41].

To exploit biomolecules in photovoltaic devices, it is critical to figure out the interaction of biomolecules with semiconductors at the molecular level. For most of the cases, biomolecules are deposited above or below the semiconductor layer, so many different molecular interaction mechanisms can occur at the bio-interface, that govern the performance of the resulting device. These interactions are mediated through specific chemical and physical bonding processes that influence both the structural orientation and electronic behavior of the biomolecule–semiconductor interface. For example, in perovskite solar cells, chemical groups such as carboxyl, phosphate, and amine groups, typically present in proteins, DNA, and polysaccharides, can form coordination bonds with metal oxide surfaces, or perovskite absorbers, improving the charge transfer from a layer to the next one, as well as resulting in enhanced durability by a passivation of the surface defects of the semiconductor. The specific functional groups present in organic molecules can interact with surface ions on the perovskite film, thereby modulating the crystallization process and concurrently repairing and passivating existing defects within the perovskite layer [42]. Also, electrostatic interactions, such as those between negatively charged phosphate backbones in DNA and positively charged surface sites, can further stabilize molecular arrangements and impact charge separation dynamics [43].

Furthermore, electrostatic interactions, arising from the overall charge distribution of biomolecules and surface charge states, can dictate adsorption orientation and interface stability. For example, when biomolecules such as DNA are introduced during perovskite film formation, the negatively charged phosphate backbone of DNA electrostatically interacts with positively charged metal ions (e.g., Pb^2+^) on the perovskite surface. This promotes an ordered and stable adsorption of DNA, improving the control over crystallization and stabilizing the interface between the biomolecule and the semiconductor [44]. These mechanisms collectively determine electron mobility, recombination rates, and ultimately, photovoltaic performance at biohybrid junctions.

Covalent bonding can occur via functional groups such as carboxyl, amine, or thiol groups as well, which can form stable chemical linkages with metal oxide surfaces like TiO_2_ or ZnO [45,46]. Hydrogen bonding is also exploited, especially when hydroxyl or amine groups in biomolecules align with surface –OH terminations on semiconductors. These interactions can facilitate organized self-assembly while influencing electronic coupling. Similarly, π–π stacking between conjugated biomolecule, such as porphyrins and hybridized carbon substrates like graphene or carbon nanotubes can promote efficient charge transfer by enhancing orbital overlap [47].

Moreover, biomolecules are derived from renewable and biodegradable sources, making them more environmentally friendly and sustainable. This aspect makes them attractive for their large-scale use as passive materials in solar cells, as well. In particular, on this point of view, materials derived from plants such as plant fibers, paper, wood, and textiles, as well as emerging forms of nanocellulose, exhibit properties well-suited to various photovoltaic applications as eco-friendly supports and sustainable encapsulating materials [48].

This adaptability underscores the potential for renewable, plant-derived materials to meet a broad range of technical requirements in sustainable solar energy systems [49]. These alternative materials have significant environmental and ecological advantages towards reduction of the overall PV carbon footprint. Their biodegradable nature ensures minimal long-term environmental impact, aligning perfectly with the objectives of sustainable energy development. By incorporating these green materials, the production of solar technologies becomes cleaner and more aligned with global efforts to transition to renewable energy sources [50]. Table 1 provides an overview of conventional photovoltaic materials and the roles played by biomolecules, focusing on critical parameters such as power conversion efficiency, long-term stability, production cost, and ecological impact.

**Figure 4:**
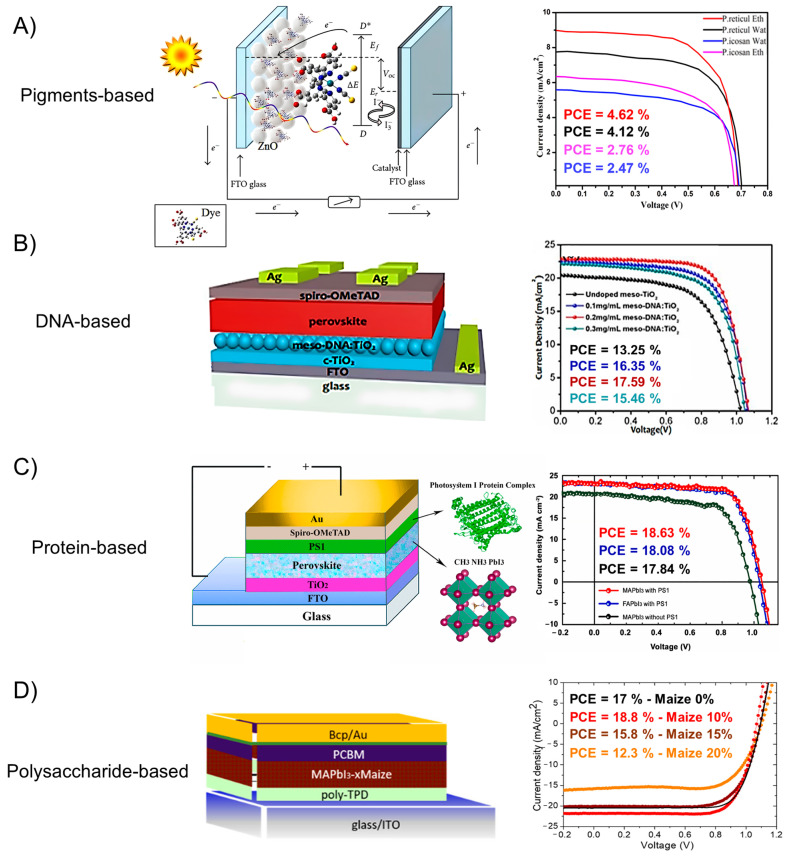
Representative examples of solar cells incorporating biomolecules to enhance performance (**A**) dye-sensitized solar cell using natural dyes, showing different PCE depending on the dye source [72], D indicates the dye molecule and D* stands for the dye excited state, (**B**) perovskite solar cell where mesoporous TiO_2_ is doped with DNA to improve light harvesting and charge transport, resulting in enhanced PCE [73], (**C**) hybrid bio-perovskite solar cell integrating Photosystem I (PSI) protein complexes to exploit natural photosynthetic mechanisms and improve efficiency [63,74], and (**D**) perovskite solar cell in which maize-derived biomolecules are blended into the MAPbI_3_ layer, tuning the film morphology and boosting photovoltaic performance [63].

The use of biomolecules has generally been directed along two main paths: light harvesting and PV applications. Inspired by the idea of emulating natural photosynthesis, several studies were pursued with the goal of developing sustainable and cost-effective methods for solar energy capture. By studying natural systems, scientists can better understand the efficient mechanisms of light capture and energy transfer at the molecular level. The way biological systems capture solar energy is impressive and can inspire the design of biohybrid devices. Photosynthesis begins with the absorption of light followed by rapid chemical reactions and electron transport processes. Sunlight is captured by thylakoid membrane proteins known as light-harvesting complexes (LHCs), with LHCII being the most abundant. These complexes serve two crucial functions: under low light conditions, they absorb and transfer solar energy to the reaction center, while in high light conditions, they protect the plant by dissipating excess energy as heat through a process called non-photochemical quenching [71]. These principles have provided natural models to inspire the development of innovative optoelectronic technologies. In the following sections, an overview is provided of the use and recent advancements of different categories of biomolecules, such as pigments, DNA, proteins, and carbohydrates, in light-harvesting systems and solar cells, with particular emphasis on the role these biomolecules play in the fabrication and operation of the reported systems. Some relevant examples of interesting results obtained with solar cells integrated with biomolecular additives are reported in Figure 4, showing the potentiality of biomolecules in improving the performance of solar devices at different levels by modifying the physical and chemical properties of the absorber as well as of the charge transport layers.

### 3.2. Pigments and Natural Small Molecules

The emulation of natural photosynthesis is central to advancing photovoltaic research, with the goal of developing sustainable and cost-effective methods for solar energy capture. The first step in this process was to exploit the smallest building blocks Nature uses to carry out the photosynthetic chemical reactions, which are pigments. Natural pigments are fundamental components of the photosynthetic machinery, playing essential roles in light absorption and energy conversion across plants, algae, and cyanobacteria. They can be thought of as being integrated in modern light-harvesting systems.

#### 3.2.1. Light Harvesting

By emulating their function in photosynthetic natural systems, pigments have been integrated in artificial light-harvesting systems in order to catch light and trigger photochemical reactions. The way biological systems capture solar energy is impressive and can inspire the design of biohybrid devices [75]. Thanks to their absorption and tunability, biomolecules exhibit an extraordinary ability to self-assemble into highly organized, hierarchical structures. This self-assembly mirrors the sophisticated arrangements found in natural light-harvesting systems, such as those in photosynthetic organisms. These well-ordered structures naturally facilitate the efficient movement of electrons, creating optimized pathways that are critical for effective energy conversion. By replicating these natural systems, biomolecular materials can significantly improve the performance of artificial light-harvesting devices. In addition, the photosynthetic efficiency of pigments used in these systems can be further improved by tailoring their absorption range, chemical stability, and photophysical properties, thereby optimizing their role in artificial light-harvesting applications [76]. For example, light-harvesting proteins chemisorbed at the interface of the semiconductor can enhance the photo responsiveness of the electrodes, resulting in a higher yield of the photoelectrochemical cell (PEC) [77]. Many studies utilize phycobiliproteins (PBPs), which are pigment-proteins found mainly in red algae and cyanobacteria. They contain phycobilins, such as phycoerythrin and phycocyanin, which absorb light at wavelengths not captured by chlorophyll, expanding the light spectrum available for photosynthesis [78]. Seven types of natural and artificial PBPs have been tested in DSSCs as sensitizers to capture low light underwater [57].

#### 3.2.2. Light Absorption and Charge Separation in Photovoltaics

The integration of pigments and natural small molecules in solar devices, and especially in third-generation solar cells, may provide multifold advantages. These molecules may improve film morphology and stability, reducing defects and passivating surfaces, protecting the active layers from environmentally induced degradation, but also improve light absorption or can be used as layers for charge carrier transport [79]. The wide array of photosynthetic pigments, each with unique absorption spectra, offers opportunities to engineer plants for specific environmental conditions or to enhance productivity by broadening the spectrum of light absorption, thus maximizing the amount of usable light. Therefore, in solar cell applications, likely the most important function played by those molecules is light absorption and charge separation. Among the photosynthetic pigments, key molecules include carotenoids, flavonoids, and chlorophyll. Carotenoids function not only in light capture but also serve as critical antioxidants, protecting the system from photodamage and photoinhibition [71]. Flavonoids, easily extracted from various plant parts such as roots, stems, leaves, flowers, and fruits, have shown significant potential in photovoltaic applications due to their advantageous optical properties. Their color, which can be tuned [80], further enhances their utility in this field. For example, anthocyanins, the most abundant flavonoid pigments, offer broad absorption spectra in the visible range and have been effectively anchored to mesoporous metal oxide electrodes via carboxylic and hydroxyl groups. By this chemical modification, a maximum of 0.390% was reached at pH 8. Notably, the solar conversion efficiency showed a significant increase of 46.62% respect to the unfunctionalized control that can be associated with the improved interaction between the dye and the TiO_2_ surface. This enhanced interaction likely promotes stronger dye anchoring, and reduced interfacial recombination losses [81].

Similarly, betalains, another class of pigments, exhibit favorable properties such as broad visible absorption and short excited-state lifetimes, which have also been successfully applied in DSSCs. In particular, DSSC incorporating a betanin-sensitized film achieved a maximum photocurrent density of 2.42 mA/cm^2^ and an open-circuit voltage of 0.44 V when using methoxypropionitrile with an I^−^/I_3_^−^ redox mediator, reaching a maximum of 0.4% of PCE at pH 8. For comparison, photocurrent and photovoltage values were also measured for DSSCs sensitized with yellow betaxanthin and a brown betalain-derived oxidation product. Although the efficiencies reported for this system are lower than 0.2%, promising values were observed for the wavelength-dependent incident photon-to-electron conversion efficiency measurements, which revealed peak values of 14% for betaxanthin-based cells and 8% for betanin-based cells, thus providing important preliminary data for optimizing the use of these molecules in solar cells [52,82].

Organic dyes possess unique properties that can be exploited to produce active layers in hybrid photovoltaic cells [83]. Among them, Porphyrins, naturally occurring macrocyclic compounds characterized by a ring structure with a metal-binding central cavity, have been widely studied.

These molecules are the functional moiety of chlorophylls, absorbing light across a range of wavelengths, enabling photosynthetic organisms to adapt to varying environmental conditions and transient light changes fundamental for photosynthesis [84]. Their electronic spectra display two distinct absorption regions: the first in the range (250–500 nm) and the other in the region (550–750 nm). The electronic structures and excitation properties of these dyes, along with their photon-to-current efficiencies, have been extensively studied in relation to the possibility of integrating them in DSSC [50,85,86]. Numerous porphyrin-based dyes have been developed, incorporating electron-donating and electron-accepting groups to extend their π-system, enhancing light absorption and improving efficiency [87]. Co(II) and Co(III)-based porphyrin compounds were efficiently used as hole transport layers in perovskite based cells in devices were Sinapoyl malate, a natural plant sunscreen molecule which protects leaves from harmful ultraviolet radiation was used to help perovskite based solar cells sustaining long term UV illumination while enhancing the electron extraction and reducing recombination phenomena at the interface.

The introduction of manganese porphyrin between a perovskite photoactive phase and TiO_2_ electron transport layer increased the formation of larger perovskite grains, facilitating electron transport to the electrode. Furthermore, this layer, due to its hydrophobic character, improved the durability and stability of the cells [88]. Modified porphyrins were used as photoactive material in organic solar cells, resulting in the optimization of the device performances, also facilitating the production process by avoiding the use of high-boiling-point chemical additives or post-treatments [53].

Peng and coworkers presented photovoltaic fibers utilizing organic dyes and their waveguide-based photovoltaic modules, obtaining devices that achieve energy conversion efficiencies above 3% by optimizing dye adsorption and reducing charge recombination at interfaces. The combination of organic dyes with luminescent solar concentrators (LSCs) could lead to high power output with narrow-band absorption sensitizers, offering promising potential for building-integrated photovoltaic applications [51].

#### 3.2.3. Solar Cells Stabilization and Durability

A further interesting example of a rational strategy to exploit the use of biomolecules in solar cells was as passivating agents to improve durability of photoactive synthetic materials. To this aim, recently the use of nicotinamide in PV devices has been shown [89]. The study highlighted the crucial role of the carbonyl group, particularly when combined with a hydrophobic structure of proper length. This combination enables the saturation of typical surface defects of the active phase, enhancing its stability over time and protecting it from external chemical degradation factors such as water and oxygen. In the same work, many different cell parameters were evaluated, even the cost related to the use of the biomolecular additives, highlighting in some cases an interesting combination of improved efficiency and cost-effectiveness for nicotinamide. In another study, four amino acids, such as glycine (Gly), glutamic acid (Glu), proline (Pro), and arginine (Arg), were selected as prototype materials to investigate their passivation effects on methylammonium lead triiodide (MAPbI_3_) perovskite films. The study focused on how their varying abilities to coordinate with Pb^2+^ ions influence the electronic quality of the perovskite and its photovoltaic performance. The results showed that the interaction strength between the amino acids and undercoordinated Pb^2+^ ions directly affects the electronic properties of the film. Among them, arginine (Arg) demonstrated the most effective passivation, attributed to its guanidine functional group, which forms strong coordination bonds with Pb^2+^. As a result, PSCs treated with arginine and fabricated using a blade-coating method achieved a high PCE of 20.49% [90]. Moreover, studies have also demonstrated that the performance limitations of zinc oxide (ZnO), commonly used as an ETL in inverted OSCs, can be mitigated by incorporating a low-cost, environmentally friendly biomolecule, as potassium aspartic acid (PAA), used as a passivating interlayer. This strategy significantly enhances charge transport and stability, leading to notable improvements in device efficiency [91].

### 3.3. DNA-Based Nanostructures

In recent years, the use of DNA-based materials has been explored since DNA macromolecule offers several key advantages, such as adjustable length and self-assembly capabilities, making it an ideal material for nanoscale applications [92,93]. DNA is a polyanion copolymer of nucleotides that holds the genetic information essential for all life forms and it was suggested to have promising electronic transport properties [94]. It is composed of nucleotides, which consist of three components: a nucleobase (cytosine [C], guanine [G], adenine [A], or thymine [T]), a deoxyribose sugar, and a phosphate group [95]. These nucleotides are connected by covalent bonds between alternating sugar and phosphate groups. The double helix structure of DNA involves two strands coiling around each other, stabilized by hydrogen bonds and base-stacking interactions between the nucleobases. Each base pair rises approximately 3.4 Å along the helix axis [96,97]. Recent research has revealed additional DNA structures beyond the well-known double helix, including triple helices and G-quadruplexes, expanding our knowledge of DNA’s structural diversity and self-assembly properties [93]. Main focus of the research in this field is on the possibility of engineering DNA strands and precisely assembling them into virtually any desired nanostructure using DNA, DNA origami, or origami-like techniques [98]. Thus, mimicking natural photosynthetic systems, which exploit large networks of chromophores, accurately organized DNA was used as a template to guide the desired spatial organization and ordering of components in organic solar cells, both for light harvesting [99] and photovoltaic devices [100]. Even as, under UV illumination, DNA molecules per se may function as light–harvesting systems, with their π-stacked bases enabling charge and energy transport [101].

Several different mechanisms of charge transport in DNA have been proposed and experimentally studied [102,103]. In particular, charge transport in DNA is generally understood to occur via a multi-step hopping process. The base stacking creates a one-dimensional π-electron system along the helical axis, which can facilitate charge movement under certain conditions. In this mechanism, charges move from one base to another. The efficiency of this process is strongly influenced by the sequence, presence of mismatches, and thermal fluctuations [104].

In certain configurations, particularly in dry, well-ordered films or DNA nanostructures, some degree of charge delocalization may occur across stacked bases [105].

The charge transport in DNA is also significantly affected by its environment.

Water molecules, counterions (such as Na^+^ or Mg^2+^), and the surrounding matrix can influence DNA conformation and modulate the energetic landscape of charge transport. Some models describe environment-assisted hopping or diffusion-controlled movement, where hydration and ionic interactions play a crucial role in enabling or hindering charge mobility.

These different mechanisms can coexist or dominate under different experimental conditions and structural configurations.

In the context of light harvesting and photovoltaic applications, these charge transport properties are particularly significant. For example, intercalating pyrrole-imidazole polyamides with selected optical properties into DNA duplexes resulted in an efficient photonic wire capable of charge transport over distances exceeding 20 nm. The choice of fluorophores and the optimization of interfluorophore distances were key to achieving efficient energy transport [106].

Photonic wires were also fabricated using DNA origami techniques and used as efficient light-harvesting systems. star-shaped DNA nanostructures [99], were used to control light energy at the nanoscale, transferring it through controlled energy transfer along a series of precisely positioned chromophores, thereby creating a large-scale photonic network [107].

#### DNA for Improved Charge Transfer

**Figure 5:**
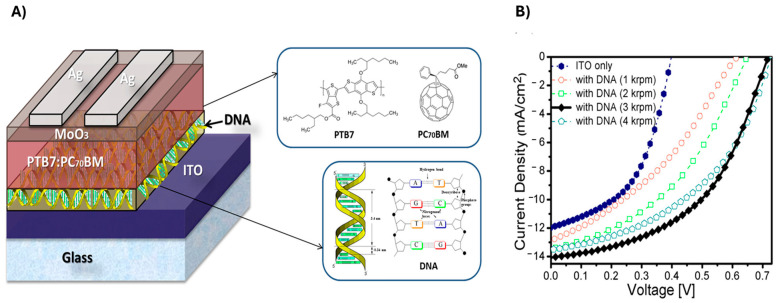
(**A**) Schematic illustration of the ITO/DNA/PTB7:PC_70_BM/MoO_3_/Ag device structure (left) and insets (right) showing the chemical structure of PTB7, PC_70_BM, and a generic strand of DNA, (**B**) J-V characteristics under AM1.5G illumination (1000 W/m^2^) for ITO/DNA/PTB7:PC_70_BM/MoO_3_/Ag polymer solar cells incorporating DNA electron extraction layers of varying thicknesses [55].

DNA can be properly introduced in PV devices as an interlayer to facilitate and improve charge transfer processes. Indeed, DNA is characterized by favourable energy levels for charge carriers transport, which, combined with excellent chemical and thermal stability, make it a promising material for photovoltaics. Noteworthy, DNA thin films can be safely conditioned at temperatures about 100 °C, to remove residual water, then avoiding the detrimental introduction of water molecules in the PV devices [108]. As an example of this kind of application for DNA, ZnO films coated with DNA presented an increased PCE with respect to the same system in the absence of DNA, thanks to the improved electron transfer from ZnO to the electrode [54]. Dagar et al., demonstrated that incorporating DNA into polymer solar cells significantly improved performance, achieving a PCE of 5% [55] (Figure 5). Further enhancements could be achieved by improving the uniformity and coverage of the DNA film on the ITO surface, as well as optimizing its electronic properties through methods such as solvent engineering, temperature treatments, or the use of additives. Additionally, modifying DNA with different cations or functional groups, along with enhancing the morphology of the photoactive blend, could further boost electron extraction efficiency. Modified DNA was introduced in perovskite solar cells both as a bio-based charge transport layer [56] or incorporated into the perovskite nanocrystalline films, both improving efficiency and stability of the device. In particular, the organization of hydrophobic molecules around the perovskite grains in the DNA-modified device contributed to sustained PCE performance over a longer period compared to the pristine device.

In another study, Peng et al. have introduced various concentrations of DNA as dopant in mesoporous titanium dioxide (meso-TiO_2_) to obtain DNA-doped meso-TiO_2_ as an electron transport layer (ETL) using a hydrothermal method. The addition of DNA was found to greatly improve the surface quality of meso-TiO_2_ and significantly reduce the carrier recombination rate. Experiments with different DNA concentrations revealed that a concentration of 0.2 mg/mL resulted in the highest photoelectric conversion efficiency of 17% for the PSCs [73]. Furthermore, it has also been demonstrated that DNA can direct the assembly of fullerenes in aqueous solution, as studied through UV/Vis absorbance and circular dichroism (CD) spectroscopy. When incorporated into DNA-based solar cells, fullerenes exhibited improved external quantum efficiency, likely due to stronger fluorescence quenching and enhanced exciton dissociation. To further improve photon-to-electron conversion efficiency, future efforts could focus on template-specific binding to optimize the percolation pathways of charge carriers toward the electrodes. This highlights DNA’s potential as a structural element for organizing chromophores into functional π-systems, which could be employed in future organic optoelectronic devices [109].

### 3.4. Proteins for Energy Conversion: From Natural Photosynthesis to Bio-Inspired Photoelectrochemical Systems

Proteins are macromolecules composed of one or more polypeptides that fold into specific three-dimensional structures, enabling them to perform a wide range of functions in living organisms. These functions include catalysis, cell signaling, structural support, signaling molecules transport, and many others. Proteins also interact with small molecules, such as cofactors, and other proteins to perform complex tasks, including photosynthesis. Since photosynthesis is the largest and most efficient natural biochemical process for converting solar energy into chemical energy, it has inspired the design of some innovative artificial PV systems [39,110,111]. Owing to their distinctive physicochemical characteristics, proteins, similarly to other classes of biomolecules, have been investigated as functional components in the development of both light-harvesting and photovoltaic systems.

#### 3.4.1. Proteins for Light Harvesting

**Figure 6:**
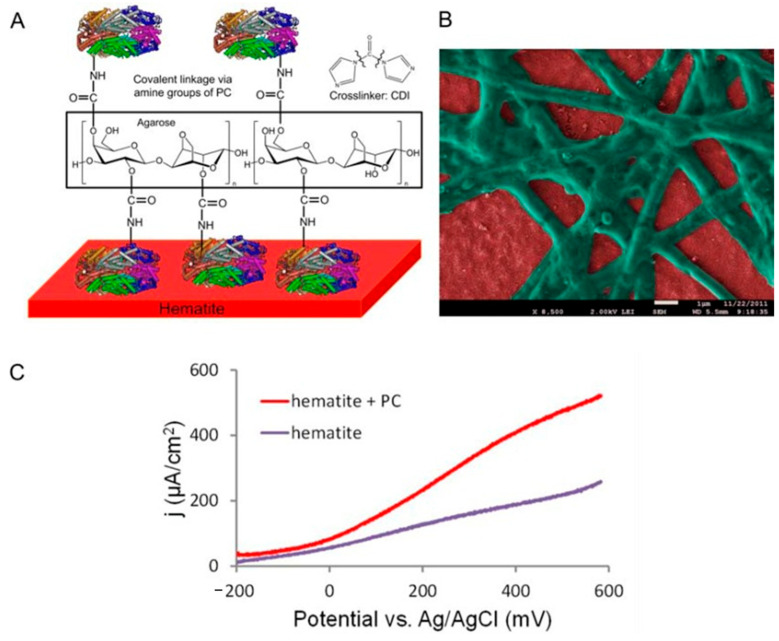
Bioengineered hematite PEC cell generated by chemical crosslinking (**A**) Scheme of hematite thin film functionalization with PC by agarose-CDI-crosslinking. (**B**) Colored SEM micrograph of a PC-coated hematite film; green: PC, red: hematite surface. (**C**) Photocurrent density enhancement achieved by PC functionalization in a hematite PEC cell; illumination: simulated solar light (AM 1.5, 100 mW cm^−2^) scan rate: 50 mV s^−1^, electrolyte: 1 M KOH [113].

Light-harvesting proteins are specialized biological complexes that capture solar energy with high efficiency and transfer it to reaction centers, playing a fundamental role in natural photosynthesis and inspiring the design of biomimetic systems for solar energy conversion. In the context of light-harvesting proteins were mainly introduced to functionalize surfaces used in PEC cells applied in water splitting for producing solar fuels, such as hydrogen. In fact, in PEC cell, a semiconductor is immersed in an electrolyte solution, forming a space-charge region. Upon illumination, photons with energy greater than the semiconductor’s band gap generate electron-hole pairs, which are separated by the electric field within the space-charge region. For photoanode, holes are driven to the semiconductor surface, where they participate in the water oxidation reaction to produce oxygen, while the photogenerated electrons are transported through the semiconductor bulk to the cathode, driving the water reduction reaction to generate hydrogen. Conversely, for a photocathode, electrons are driven to the semiconductor surface to facilitate water reduction and produce H_2_, while holes are transferred to the anode to complete the oxidation reaction and generate O_2_ [17]. In such a system, light-harvesting proteins chemisorbed at the interface of the semiconductor can enhance the photo responsiveness of the electrodes, resulting in a higher yield of the PEC cell [112]. An interesting example of this approach has been reported by Ihssen et al., who provided an overview of how electrode surfaces, particularly hematite photoanodes, can be enhanced by integrating light-harvesting proteins. They demonstrate that affordable biomaterials, such as cyanobacterial phycocyanin and enzymatically produced melanin, can significantly improve the performance of low-cost metal oxide photoanodes in PEC systems (see Figure 6) [113]. However, a significant limitation of this approach is related to the instability of proteins. Prolonged exposure to light can accelerate protein degradation due to the generation of harmful reactive oxygen species and photoinhibition [114,115]. To mitigate these challenges, researchers are developing hybrid materials that combine biological components with inorganic elements, which show promise as an effective solution.

#### 3.4.2. Proteins for Improved Light Absorption in Photovoltaics

Some relevant examples of the use of proteins in solar cells have been reported as well. PBPs, found in red algae and cyanobacteria, contain phycobilins that absorb light beyond chlorophyll’s range, enhancing photosynthetic efficiency [78]. For example, seven types of natural and artificial PBPs have been tested in DSSCs as sensitizers to capture low light underwater [57]. The DSSC sensitized with B-phycoerythrin achieved a short-circuit current density of 3.236 mA/cm^2^, open circuit voltage of 0.545 V, fill factor of 0.569, and a photoelectric conversion efficiency of 1% between 525 and 570 nm, by highlighting the potentiality of PBP-based DSSCs for underwater PV applications. Another interesting example is offered by the integration of Bacteriorhodopsin (bR) in perovskite solar cells [59]. bR has been tested at the interface with MAPI perovskite photoactive phase to enhance efficiency via Förster Resonance Energy Transfer (FRET). FRET, which occurs due to similar optical gaps between bR and perovskite, facilitates efficient exciton energy transfer. Titanium dioxide, used as ETL in the device, was functionalized with bR, which accelerates electron injection from excitons in the perovskite layer, improving photovoltaic performance. Solar cells with TiO_2_/bR layers show significantly higher efficiency compared to baseline cells, suggesting potential for developing bioperovskite solar cells with enhanced performance and stability. Further photoactive protein-based structures are Photosystems I and II (PSI and PSII, respectively). Examples of solar cells based on PSI, which acts as both a photosensitizer and charge generator. This complex is employed as the active layer in solid-state solar cells. PSI captures light through its organized antenna system composed of pigment networks. The absorbed energy is then transferred to the reaction center (RC), driving the transmembrane electron transfer process. Simultaneously, Photosystem II (PSII) absorbs light and splits water to produce oxygen while reducing membrane-bound quinones. These reduced quinones are utilized by the cytochrome b6f complex to create a proton gradient, which helps reduce PSI’s donor site, the copper protein plastocyanin (PC). Ultimately, energy flows through the PSI pigment network to P700, a special pair of chlorophyll molecules, where electrons are generated [116]. Proteins containing chlorophyll (Chl) or bacteriochlorophyll (BChl) are also interesting bio-complexes for their use in PV. In plants, Chl is bound to hydrophobic proteins within photosynthetic membranes, forming evolutionarily optimized complexes that are challenging to replicate artificially. Soluble Chl-binding proteins found in various plants organize Chl compactly within their hydrophobic cores, serving as valuable models for studying Chl interactions and designing artificial photoenergy conversion systems. A novel strategy utilizes a protein nano-ring (SR) to integrate Chl molecules, enhancing photostability and photoelectrochemical activity, which shows promise for developing advanced photoactive biomaterials. The solar cell containing the Chl a-SR complex, with approximately 18 Chl a molecules per nano-ring, achieved a PCE of 1.09%, significantly higher than other solar cells sensitized with different Chl a-SR assemblies, which had PCEs ranging from 0.43% to 0.55%. The increased efficiency of the Chl a-SR complex suggests that this specific configuration of chlorophyll molecules is more effective at converting light into electrical energy [58].

These approaches are seen as a potential path for designing biological systems for integration in next-generation optoelectronics. Indeed, peptides and proteins, while less conductive than traditional materials, possess intrinsic electrical properties that make them promising for bioelectronics. However, there are still significant challenges to address. Practical applications require improvements in performance, scalability, and stability, particularly in environments with changing temperature or pH. Progress in peptide-based bioelectronics will rely on overcoming these obstacles, with an emphasis on developing stable materials that can self-assemble for eco-friendly bioelectronics [111].

#### 3.4.3. Amyloid from Neurodegenerative Pathways to Advanced Applications in PV

Amyloid fibrils are the most stable state for protein molecules, and their unique structural characteristics suggest intriguing potential for improving solar cell performance.

Historically, research on amyloids has primarily concentrated on neurodegenerative diseases, including Alzheimer’s and Parkinson’s disease, with the goal of unraveling the mechanisms by which these fibrillar structures form and contribute to cellular toxicity [117,118]. Their unique molecular architecture consists of β-strands aligned perpendicular to the fibril axis and organized into parallel β-sheets, which makes them excellent biomaterials for many applications. These fibrils typically exhibit a cross-β motif, where β-strands stack tightly, stabilized by extensive hydrogen bonding, with an interstrand distance of about 4 Å and an intersheet distance of 10–12 Å. Despite this conserved atomic structure, amyloid fibrils display remarkable structural diversity at larger scales, forming various morphologies such as ribbons, nanotubes, nanofibrils, and 3D scaffolds [119,120]. Their structural stability is reinforced by extensive hydrogen bonding and hydrophobic interactions between β-sheets, contributing to their resistance to proteolytic degradation process is governed by intra- and inter-molecular interactions, which are influenced by the protein sequence and the physical and chemical properties of the environment [121]. A large number of applications have already been proven, ranging from photoluminescent materials, conductive nanowires, biosensors, and hybrid materials capable of mimicking biological functions [122]. This structure imparts amyloids with exceptional stability and mechanical strength. Therefore, amyloid fibrils have garnered interest for their applications in nanotechnology and biomedical fields [123]. They have been used in bio-membranes, nanodevices [124], hydrogels [125], biosensors, and energy conversion materials [126].

Amyloids exhibit interesting properties both in their bare form and as templates for the organization of other materials. In fact, it was observed that these fibrils improve the organization of inorganic materials, facilitating more efficient charge transport in photovoltaic systems, as reported for metal nanoparticles being regularly spaced along amyloid fibrils by controlling the aggregation and growth processes of the fibrils [127]. Then, the incorporation of amyloid fibrils into solar cells not only can increase device performance by controlling the materials assembly at the nano- and microscale, but it can also induce new properties directly deriving from their structural peculiarities. For example, Inganäs et al. have integrated bare amyloids of insulin into organic solar cells, resulting in enhanced transport properties. Nanofibrils influenced the organization of donor-acceptor materials, leading to significantly improved charge transport compared to systems without the fibrils [60].

**Figure 7:**
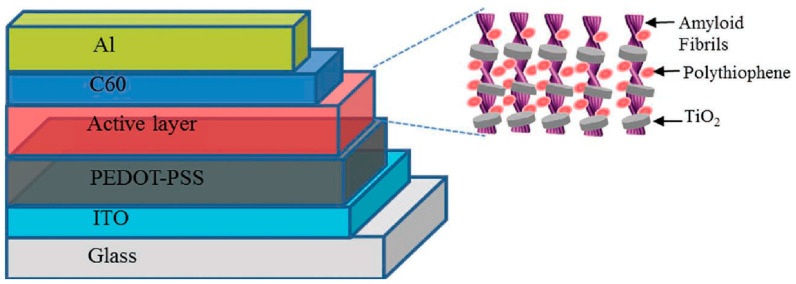
Schematic diagram of the hybrid photovoltaic device prepared using an active layer composed of the TiO_2_-hybrid nanowires blended with polythiophene [61].

On the other side, Bolisetty et al. have employed beta lactoglobulin amyloid fibrils as templates for synthesizing TiO_2_ hybrid nanowires. The fibrils, uniformly coated with TiO_2_ nanoparticles via electrostatic and hydrogen bonding interactions, were integrated into the photovoltaic active layer through spin-coating a blend of polythiophene-coated fibrils and amyloid-TiO_2_ hybrid nanowires, improving the organic solar device. The schematic of the solar cell is represented in Figure 7 to highlight the structural properties of the bio-hybrid polymeric photoactive blend tested as phase [61].

**Figure 8:**
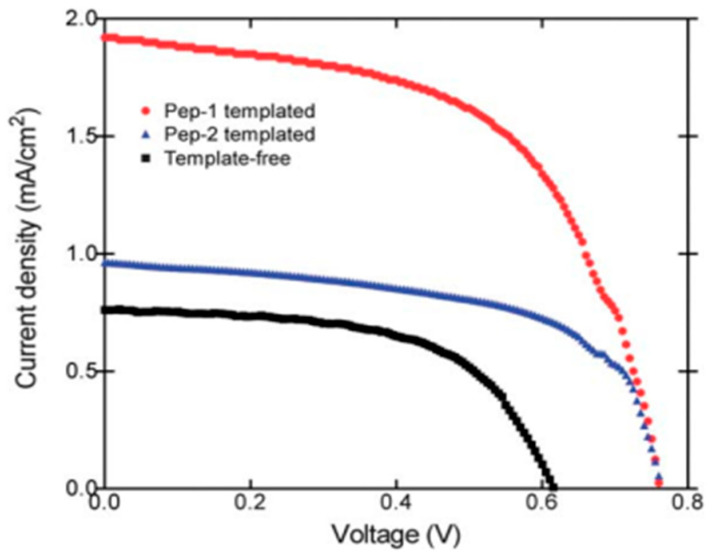
Representative J–V spectra of devices based on peptide 1 (Pep-1), peptide 2 (Pep-2) templated and template-free TiO_2_ materials [128].

Furthermore, Acar et al. utilized amyloid peptide nanofibrils to template TiO_2_ nanostructures in a bottom-up approach. After staining the calcined TiO_2_ layer with the N719 photosensitizer dye, they developed dye-sensitized solar cells with increased dye loading capacity and enhanced open-circuit voltages, benefiting from the high surface area of the amyloid fibrils [128] (Figure 8).

These studies demonstrate the potential of amyloid fibrils to enhance the performance and stability of photovoltaic devices through improved material organization and charge transport.

### 3.5. Polysaccharides

Polysaccharides are gaining attention in innovative solar cell technologies due to their renewable nature, biocompatibility, and functional versatility. They can be used as flexible or transparent supports for almost all categories of solar cells, interfacial layers, additives to tune materials properties, and encapsulating agents [129].

#### 3.5.1. Cellulosic Materials as Supports for Solar Devices

Cellulose represents the most abundant organic polymer on Earth [130]; it represents an abundant and sustainable material for electronic devices. Also, it has garnered significant research interest due to its recyclability, flexibility, lightweight nature, biocompatibility, and exceptionally low cost compared to other materials [129,131]. Interestingly, cellulose, in the form of paper, has been widely used for many electronic applications, such as solar cells, storage energy electronic devices, and batteries [131]. It can serve multiple roles within electronic devices, ranging from a flexible structural support or substrate to an active component in charge transport (e.g., paper-based electrodes) [132]. Thanks to its compatibility with printing techniques, paper also enables the integration of functional components such as sensors or batteries, serving as both a structural substrate and an active element in device operation. Additionally, its properties can be further tailored through surface modifications and functionalization, enhancing characteristics like wettability, barrier performance, and ionic transport. As an example, Gao et al. efficiently fabricated paper-based solar cells, reaching efficiencies up to 9% for opaque devices [62]. Although properties such as porosity and surface roughness can offer certain advantages, they may also pose limitations in the fabrication of photovoltaic devices. Specifically, these characteristics might hinder a proper formation of homogeneous thin films and planar junctions in PV devices, leading to a reduction in the overall efficiency of the solar cell. Nevertheless, this limitation was successfully overcome by flattening of the paper substrate through a surface metallization step, for example, with aluminum, which not only smooths the substrate but can also serve as back contact for the device [133].

An alternative strategy to address these limitations is the use of nanopaper, which offers high optical transparency due to the nanoscale organization of crystalline cellulose [134]. The fabrication of high-quality nanopaper involves processes adapted from the paper industry, such as mechanical compression under vacuum and pressure. It can then be easily integrated into factory processes for scale-up. Unlike conventional substrates, nanopaper generally does not require additional coating layers to be compatible with electronic ink deposition. Several examples of organic and perovskite solar cells fabricated on cellulose nanopaper have been reported [65,135].

Another attractive material for semi-transparent solar cells is represented by transparent wood, which has also been tested as a substrate for innovative building-integrated PV thanks to its high optical transmittance (over 85% at 1 mm thickness) and excellent mechanical properties. Perovskite solar cells were successfully fabricated directly on transparent wood substrates at low processing temperatures (<150 °C), achieving a PCE up to 16.8% [136].

#### 3.5.2. Polysaccharides for Tuning Rheology

The application of polysaccharides in solar cell technology extends beyond their role as substrates for active layers. Several studies have demonstrated their effective incorporation as additives in perovskite precursor solutions. Notably, Rizzo and co-workers reported that the addition of starches can significantly alter the rheological properties of these solutions, tuning them to meet the requirements of ink formulations compatible with scalable deposition techniques, such as inkjet printing [137]. Specifically, cornstarch has also been shown to significantly influence the morphology and surface roughness of perovskite films, enabling the formation of highly homogeneous layers with nanometer-scale roughness. By forming hydrogen bonds with the methylammonium iodide precursor, starch facilitates templated perovskite growth, resulting in a compact and uniform film deposited by spin coating in a simple one-step and antisolvent-free spin protocol. In such cases, the abundance of hydroxyl groups in the polysaccharide structure enables interactions with both perovskite precursors and solvents, influencing the solvent evaporation dynamics. This interaction leads to a slower crystallization process, which promotes the formation of less defective perovskite crystals, as observed with hydroxyethyl cellulose and cellulose acetate [138]. Notable results were reported by Giuri et al., who achieved a PCE approaching 19% for their best-performing MAPbI_3_-based device, using a precursor solution containing 10% *w*/*w* cornstarch [63].

In addition, starch has also demonstrated to provide further improvements in terms of thermomechanical and environmental stability while preserving high photovoltaic performance [63]. The improvement in the long-term stability of PSCs incorporating starch as an additive is further supported by the work of Wang et al. [64], who attributed this effect to the formation of a starch–iodine complex. This complex is capable of absorbing and storing free iodide ions commonly present in the perovskite active phase, thereby stabilizing the material through the formation of a starch–triiodide complex. Such mechanism promotes self-healing of iodide defects and suppresses iodide migration in the perovskite films, leading to improved long-term stability of the perovskite solar cells.

Further insights into the role of cornstarch in perovskite film formation were provided by Guagliardi and co-workers, who investigated its impact on crystallographic properties in FA_x_MA_1-x_PbI_3_-based compositions, including systems incorporating organic spacer salts acting as passivating agents or precursors to low-dimensional perovskites. Their study explored variations in crystalline domain size, microstrain, and crystallographic orientation (film texture) as a function of starch content across different perovskite formulations, offering a fundamental interpretation of the observations previously reported by Rizzo and co-workers [139]. The results showed a reduction in crystalline domain size accompanied by an increase in film thickness, along with a progressive randomization of crystal orientation relative to the substrate. These findings suggest that a portion of the perovskite crystals are embedded within the starch matrix, forming micrometer-sized grains. Notably, the combination of cornstarch with xylylenediammonium (XDA^2+^) yielded particularly stable formulations, pointing to a synergistic interaction between the two components. Based on these results, the authors argued that crystal orientation is not necessarily a determining factor for achieving high power conversion efficiencies, in line with the findings observed by Giuri et al. and Muscarella et al. for MAPbI_3_-based films [63,140]. Furthermore, an increase in microstrain was inferred from a starch-dependent blue shift in the perovskite optical band gap, indicating enhanced lattice inhomogeneity that might contribute to defect formation or intrinsic material instability.

Furthermore, polysaccharides can be tailored and functionalized for various purposes, including the development of multifunctional materials suitable for stable perovskite deposition under ambient conditions. Notably, they can facilitate the stabilization of the desired perovskite phase without requiring additional crystallization agents such as methylammonium chloride (MACl). In this context, Vanni et al. demonstrated the incorporation of camphorsulfonic acid-functionalized chitosan into the perovskite layer, enabling the stabilization of the α-FAPbI_3_ phase deposited through gravure printing, in air environment [141]. Such polymer establishes multiple interactions with the perovskite surface and grain boundaries, effectively passivating trap states and structural defects. This results in the suppression of non-radiative recombination pathways, as indicated by prolonged photoluminescence (PL) lifetimes and enhanced PL intensities. Devices fabricated using this strategy retained 80% of their initial efficiency after 1200 h of exposure to air without encapsulation, highlighting the potential of polysaccharide-based additives for scalable and robust perovskite solar cell technologies [141].

Very recently, the alginate-based polysaccharides have also been incorporated into PSCs at various levels, showing promise as effective additives. In this context, Lu Y. and co-workers [142] examined the impact of incorporating a biomass-derived polymer, tetrabutylammonium alginate (TBA-Alg), into the perovskite active layer, focusing on its effects on both device performance and stability. Specifically, these observed enhancements rely on the polymer dual functionality: the alginate moieties chelate uncoordinated Pb^2+^ ions, forming a robust “egg-box” structure that curtails lead leakage and minimizes associated efficiency losses; while the hydrophobic TBA^+^ alkyl chains create moisture-resistant barriers around perovskite grains, as supported by increased contact angles. Jointly with that, the occurring Lewis base–acid interactions between TBA-Alg and Pb^2+^, confirmed by shifts in ^1^H-NMR, FTIR, and XPS spectra, prolong crystallization time and suppress defect formation, leading to improved optoelectronic properties. Like other polymeric additives, TBA-Alg forms a continuous network at grain boundaries and surfaces, enhancing mechanical integrity and environmental resilience. PSCs incorporating TBA-Alg achieve PCEs of 25.01% (p–i–n) and 24.44% (n–i–p), retaining 95.5% of their initial efficiency after 2000 h in the dark and 80% after 1050 h under illumination (~60% RH). Large-area modules (~16.8 cm^2^) also attain 22.17% PCE, confirming also the scalability purposes.

#### 3.5.3. Polysaccharides for Charge Transfer

Polysaccharides have also been explored in terms of charge transfer between the active phase and the charge transport layers. For instance, alginate-based polymers have been incorporated in PSC layers, at the interface with the ETL. In the case studied by He, J. et al. [143], sodium alginate (SA) was employed to functionalize SnO_2_, yielding a distinctive nanosynapse morphology through interactions with Sn atoms, which anchored the polymer to the SnO_2_ nanoparticle surface. This modification enhanced interface contact with the perovskite layer and improved electron extraction, as indicated by reduced steady-state PL intensity. Furthermore, ultraviolet photoelectron spectroscopy (UPS) further showed better energy level alignment, with the CBM of SnO_2_-SA closely matching that of the perovskite, thus minimizing photoelectron losses, which is consistent with the steady-state PL measurements. As an overall result, lead-based PSCs incorporating SnO_2_-SA achieved a PCE of 24.11% with a high FF of 0.835. Devices also showed enhanced stability, retaining full efficiency after 2000 h of storage, while unencapsulated cells maintained 85% of their initial performance after 700 h of continuous simulated sunlight illumination.

## 4. Summary and Outlooks

Recent advancements in the use of biomolecules have significantly opened new insights for different technologies. The incorporation of self-assembling biomimetic peptides, amyloid fibrils, and other bio-inspired materials has led to improvements in charge transport, material arrangement at the molecular level, and increased stability in PV devices. These materials not only offer remarkable biocompatibility but also pave the way for more sustainable and renewable energy solutions. Additionally, the integration of natural components, such as DNA, natural pigments, and light-harvesting complexes, has introduced new avenues for boosting the efficiency of solar cells. DNA-based materials, for example, have demonstrated the ability to self-assemble into highly ordered structures playing the role of molecular template, which can enhance and modulate charge transport and light absorption. Natural pigments, such as chlorophyll and carotenoids, have shown great potential in mimicking the light-harvesting processes of photosynthetic organisms, enabling the capture of a broader spectrum of light and improving overall energy conversion efficiency. Some polysaccharides, such as starch, have already gained popularity as additives in perovskite precursor inks, to tune rheological ink properties resulting in improved film morphology and enhanced device stability and performance. Nonetheless, several challenges still remain that hinder the widespread adoption of biomolecules in modern solar energy technologies. The scalability of these biomolecular materials for large-scale production remains a major challenge, as the processes involved in extraction, purification, and incorporating them into solar cells can be complex. Indeed, the processes involved in integrating these materials into PV systems need further refinement to ensure cost-effectiveness and feasibility for industrial applications. Additionally, the long-term stability and durability of biomolecular materials, especially in outdoor environments exposed to moisture, temperature fluctuations, and UV radiation, lead to material degradation, limiting their lifespan and reliability, and require further investigation, especially if the folding has to be maintained for the biomolecules to perform their function in the device. Moreover, while natural pigments and DNA-based materials show promise, their efficiency in energy conversion still lags behind that of traditional inorganic materials, and further optimization is necessary to match their performance. Moving forward, further research into the optimization of these biomolecular materials, along with their integration into large-scale production systems, will be crucial in unlocking their full potential. Addressing these limitations opens exciting opportunities for future research and development. Developing protective coatings or hybrid materials that combine biomolecular and inorganic components may improve durability and resistance to environmental stressors. Research into encapsulation techniques and UV stabilizers will be crucial. Continued exploration of molecular structures and synthetic biology can enhance the efficiency of bio-inspired materials. For instance, genetic engineering of natural pigments or peptides may yield materials with superior light-harvesting capabilities and improved charge transport. Collaborations between material scientists, biologists, and engineers will be key to translating laboratory advancements into practical solutions. Continued innovation in this field promises to drive the development of more efficient, cost-effective, and eco-friendly solar energy technologies, making a significant contribution to the global transition towards renewable energy.

## Figures and Tables

**Table 1:** Comparative overview of conventional photovoltaic materials and biomolecular counterparts with respect to economic viability, operational stability, power conversion efficiency, and environmental sustainability.

Material Type	Device Type	Function	PCE (%)	Cost	Stability	Sustainability	Refs.
**Biomolecules**							
**Pigments**	DSSC, OSC, PSC	Sensitizers, Light absorber, Improves charge transport	~2–18	Low	Moderate (photo-degradable)	High	[51,52,53]
**DNA**	OSC, PSC	Template for layer organization, Charge transport	~5–15	Low–Moderate	High (sensitive to UV)	High (biocompatible, renewable)	[54,55,56]
**Proteins**	DSSC PSC OSC	Structural scaffold	~0.34–14	Low–Moderate	Low–Moderate	High	[57,58,59,60,61]
**Polysaccharides**	PSC OSC	Encapsulant	~3–22	High	High	Very High (biodegradable, abundant)	[62,63,64,65]
**Tradizional Materials**							
**Silicon**	Si-PV	Light absorber	20–27	High	Very High (25+ years)	Low (energy-intensive fabrication)	[66]
**Perovskite (e.g., MAPbI_3_)**	PSC	Light absorber	22–25	Moderate	Moderate	Low	[67]
**P3HT/PCBM**	OSCs	Charge transport	~3–5	Moderate	Moderate (photochemical degradation possible)	Moderate	[68]
**Ethylene vinyl acetate (EVA)**	Si-PV PSCs	Encapsulant	20–25	Moderate	Moderate	Moderate	[69,70]

## Data Availability

No new data were created or analyzed in this study. Data sharing is not applicable to this article.
